# Isolation, draft genome sequence, and identification of *Paenibacillus glycanilyticus* subsp. *hiroshimensis* CCS26

**DOI:** 10.7150/jgen.87229

**Published:** 2023-09-25

**Authors:** Hironaga Akita, Yoshiki Shinto, Zen-ichiro Kimura

**Affiliations:** 1 College of Industrial Technology, Nihon University, 1-2-1 Izumi-cho, Narashino, Chiba 275-8575, Japan.; 2 Department of Civil and Environmental Engineering, National Institute of Technology, Kure College, 2-2-11 Aga-minami, Kure, Hiroshima 737-8506, Japan.

**Keywords:** *Paenibacillus*, Oligotroph, 16S rRNA, Draft genome sequence, Average nucleotide identity

## Abstract

To isolate the useful strain for fermentation to produce bioactive compounds, we screened oligotrophic bacteria, and then strain CCS26 was isolated from leaf soil collected in Japan. This strain was capable of growth on low-nutrient medium. To elucidate the taxonomy of strain CCS26, we determined the 16S rRNA gene and draft genome sequences, respectively. A phylogenetic tree based on 16S rRNA gene sequences showed that strain CCS26 clustered with *Paenibacillus* species. The draft genome sequence of strain CCS26 consisted of a total of 90 contigs containing 6,957,994 bp, with a GC content of 50.8% and comprising 6,343 predicted coding sequences. Based on analysis of the average nucleotide identity with the draft genome sequence, the strain was identified as *P. glycanilyticus* subsp. *hiroshimensis* CCS26.

## Introduction

Fermentation is used in industrial applications to produce bioactive compounds such as antibiotics, vitamins, and amino acids. For example, *Corynebacterium glutamicum* has been used in amino acid production worldwide for more than 50 years, and approximately 3 million tons of L-glutamate were produced using *C. glutamicum* in 2014 1. These industrial applications involve the preparation of culture media, fermentation of the target compound, and separation and purification of the compound. As the process of fermentation is complex, the production cost of fermented compounds is higher than that of chemically synthesized products in many cases. Thus, fermentation with low production costs is important for industrial applications.

One promising approach for reducing production costs is the utilization of oligotrophs that are unaffected by a high-nutrient condition. Oligotrophs can grow under low-nutrient conditions. Several kinds of oligotrophs are isolated from soil, rivers, lakes, and oceans lacking organic substances 2, however most oligotrophs are inhibited under high-nutrient conditions. Consequently, oligotrophs have not been applied for industrial use. Previously, we developed a simple method for the isolation of oligotrophs that can grow under high-nutrient conditions without growth inhibition. In our method, efficient isolation of objective oligotrophs is possible by screening using 1.5% agar plates, novel bacteria such as *Deinococcus kurensis* KR-1^T^ 3, *Enterobacter oligotrophicus* CCA6^T^ 4, *Pseudomonas humi* CCA1^T^ 5, and* Paenibacillus glycanilyticus* subsp. *hiroshimensis* CCI5^T^ 6 are isolated. In this study, we screened oligotrophs that increase the cost-effectiveness of fermentation processes, and isolated a novel oligotroph, strain CCS26, which exhibits the potential to enhance fermentation efficiency. The genome of strain CCS26 was sequenced in order to facilitate future in-depth genomic studies and industrial applications of this strain.

## Materials and Methods

Based on our previous studies 3-6, soil samples including compost, leaf soil, mud, and peat moss, were collected at Narashino City in Chiba Prefecture, Japan. Oligotrophs were isolated on 1.5% agar plates (pH 7.2) containing sulfate (<0.4%), calcium (<0.1%), iron (<0.01%), and a few fatty acids and/or other minerals (chlorine, iodine, potassium, zinc) at concentrations of less than 0.01%. Soil samples were suspended separately at 10% (w/v) with sterilized water and then filtered using Advantec filter paper No.1 (size: 90 mm; Toyo Roshi) to prepare suspensions. The filtrates were inoculated onto 1.5% agar plates and then incubated for 2 days at 37°C. Individual colonies that grew on the plates were successively re-streaked onto fresh 1.5% agar plates at least three times to obtain pure colonies, and the pure colonies were used for phylogenetic characterization.

To extract genomic DNA, strain CCS26 was cultured at 37°C for 2 days in R2A broth (Nihon Pharmaceutical), and the cells were harvested by centrifugation and washed twice with sterile water. Genomic DNA was extracted from the culture using an Illustra^TM^ bacteria genomicPrep Mini Spin kit (GE Healthcare, Chicago, IL, USA). The concentration and purity of the resulting genomic DNA were determined using a Quant-iT dsDNA Assay Kit (Invitrogen) and NanoDrop ND-1000 spectrophotometer (Thermo Fisher Scientific), respectively.

Using the genomic DNA as a template, the 16S rRNA gene was amplified using KOD-plus DNA Polymerase (TOYOBO, Osaka, Japan) with the bacterial universal primers 27f 7 and 1391r 8. Subsequently, the amplified PCR product was purified using a Wizard SV Gel and PCR Clean-Up System (Promega, Madison, WI, USA). The purified product was cloned into the pTA2 vector (TOYOBO), and the resulting vector was sequenced. The sequence of the 16S rRNA gene (accession number: LC763768; 1218 bp) was compared with reference sequences available in the GenBank/EMBL/DDBJ databases using BLAST. Multiple alignment and construction of a maximum-likelihood tree were performed using MEGA-X 9 with the Tamura-Nei model 10.

Libraries for genome sequencing were prepared using a Nextera XT DNA Library Preparation Kit (Illumina, San Diego, CA, USA). The resulting libraries were sequenced using a MiSeq sequencer (Illumina) and MiSeq Reagent Kit v3 (Illumina). Default parameters were used for all software unless otherwise specified. Quality control and* de novo* assembly were carried out using Trimmomatic ver.0.39 11 and Shovill ver.1.1.0 12, respectively. Genome annotation was carried out using DFAST ver.1.2.0. 13. Average nucleotide identity (ANI) values were calculated by pairwise comparison of the genome sequences of strain CCS26 and the related type strains using the ANI algorithm 14 implemented within OrthoANIu tools 15.

## Results and Discussion

When each filtrate was plated on a 1.5% agar plate without a carbon source and other medium components, a single colony was obtained only from a sample of leaf soil. Using standard dilution plating on the same plates, a purified colony was obtained and designated strain CCS26.

**Figure 1:**
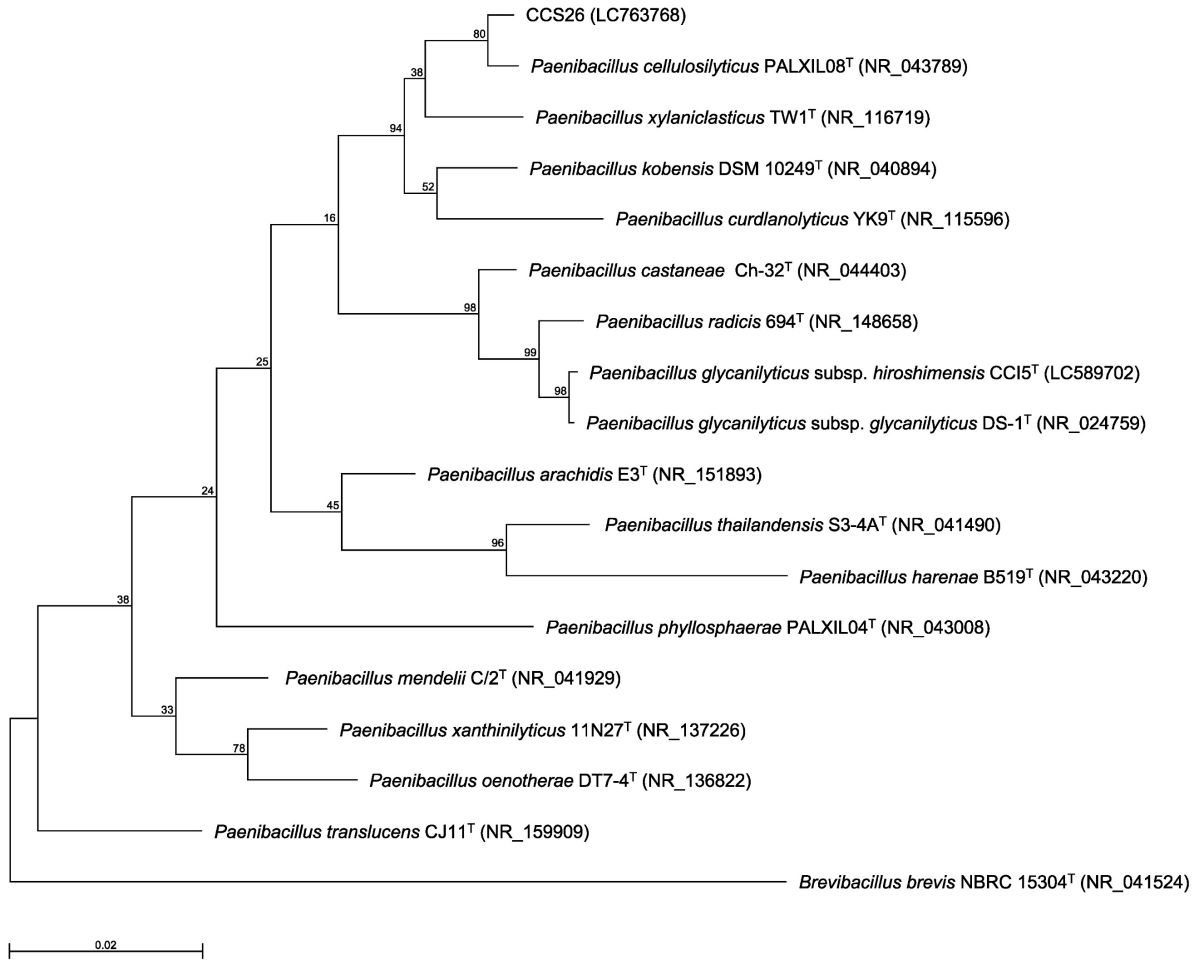
Phylogenetic tree constructed from analysis of 16S rRNA gene sequences and showing the relationships between strain CCS26 and related *Paenibacillus* type strains. The bar indicates a 0.02% nucleotide substitution rate. The tree was rooted using *Brevibacillus brevis* NBRC 15304^T^ as the outgroup.

Previously, we identified bacteria isolated from environmental soil samples as novel type strains based on the 16S rRNA gene sequence and ANI value 3-6. In our method, the genus of the isolated strain was determined by constructing a phylogenetic tree based on 16S rRNA gene sequences, and the novel species was identified based on the ANI cutoff value for prokaryotic species delineation between the isolated strain and the most closely related type strain. Thus, the 16S rRNA gene sequence was first determined. In the maximum-likelihood tree constructed based on almost complete sequences of the 16S rRNA gene, strain CCS26 clustered with members of the genus* Paenibacillus* (Figure 1). Moreover, the sequences of the following *Paenibacillus* type strains showed similarity to that of strain CCS26: *P. cellulosilyticus* PALXIL08^T^ (99.3%), *P. kobensis* DSM 10249^T^ (98.6%), and *P. curdlanolyticus* YK9^T^ (98.2%).

To elucidate the taxonomy of strain CCS26, we determined its draft genome sequence. Genome sequencing using a MiSeq sequencer yielded 148,470 reads with 47-fold coverage. The assembled genome sequence of strain CCS26 contained 90 contigs consisting of 6,924,729 bp, with a GC content of 50.8%. The largest contig and N50 contig size were 540,821 bp and 196,675 bp, respectively. Within the genomic DNA of strain CCS26, 6,340 predicted coding sequences were identified. The highest ANI value (98.3%) was confirmed between strain CCS26 and *P. glycanilyticus* subsp. *hiroshimensis* CCI5^T^ 6, which exceeded the cutoff value of 98% for prokaryotic subspecies delineation 16. Thus, strain CCS26 was identified as *P. glycanilyticus* subsp. *hiroshimensis* CCS26. Based on 16S rRNA gene sequence homology as well as physiologic and chemotaxonomic characteristics, more than 300 species and 6 subspecies have been identified in the genus* Paenibacillus* to date (https://www.bacterio.net/genus/paenibacillus). However, growth capacity under oligotrophic conditions has been reported only for *P. glycanilyticus* subsp. *hiroshimensis*, indicating that the subspecies may have unknown growth potential. Thus, we are planning to characterize the growth potential of *P. glycanilyticus* subsp. *hiroshimensis*, and the results will be described elsewhere as the next stage of our study.

### Nucleotide Sequence Accession Numbers

The draft genome sequence of *P. glycanilyticus* subsp. *hiroshimensis* CCS26 was deposited in the DDBJ/EMBL/GenBank databases under accession numbers BTCL01000001 to BTCL01000090. Raw sequence reads were deposited in the DDBJ under BioProject number PRJDB15612 and BioSample number SAMD00590492.
